# Testosterone deficiency in men receiving immunotherapy for malignant melanoma

**DOI:** 10.18632/oncotarget.27876

**Published:** 2021-02-02

**Authors:** Madeline Peters, Amy Pearlman, William Terry, Sarah L. Mott, Varun Monga

**Affiliations:** ^1^Carver College of Medicine, University of Iowa, Iowa City, IA, 52242, USA; ^2^Department of Urology, University of Iowa, Iowa City, IA, 52242, USA; ^3^Department of Pediatrics, University of Iowa, Iowa City, IA, 52242, USA; ^4^Holden Comprehensive Cancer Center, University of Iowa, Iowa City, IA, 52242, USA; ^5^Department of Medicine, University of Iowa, Iowa City, IA, 52242, USA

**Keywords:** cancer, melanoma, immunotherapy, testosterone deficiency, androgens

## Abstract

Immunotherapy has been established as a standard of care for patients with malignant melanoma, however, the long-term side-effects of immunotherapy are still emerging. Studies over the last decade have documented increasing reports of endocrine dysfunction following the initiation of immunotherapy. Our study aimed to detect the proportion of men who have low testosterone before, during, and or/after receiving immunotherapy for malignant melanoma, and to determine the proportion of men who receive testosterone replacement therapy after detection of low testosterone. We performed retrospective chart review of patients with malignant melanoma treated with immunotherapy. Low testosterone was identified in 34 out of 49 patients at some point during their treatment with immunotherapy. Despite low testosterone levels in two-thirds of patients, only three patients were treated with testosterone replacement therapy. In addition to laboratory evidence of low testosterone, patients were also symptomatic as 43 out of 49 patients reported fatigue to their providers. Four patients developed hypophysitis and subsequent hypopituitarism, all of whom were receiving Ipilimumab. We conclude that patients with stage 3 or 4 melanoma treated with immunotherapy appear to be at an increased risk of developing testosterone deficiency during their treatment.

## INTRODUCTION

Malignant melanoma is one of the fastest growing cancers worldwide and men account for 60% of new diagnoses [1]. Fortunately, cancer treatments have greatly improved and those impacted by cancer are now living longer. Immunotherapy has been established as a standard of care for patients with malignant melanoma, however, the long-term side-effects of immunotherapy are still emerging [2].

It has been well documented that cancer treatment-related toxicities create short and long-term side effects, which can significantly impair the quality of life of cancer survivors. Conventional chemotherapy agents have been thoroughly studied, and there are an abundance of data detailing the negative effects of chemotherapy on testosterone levels, fertility, and the quality of life of cancer patients [3–5]. As immunotherapy is a relatively new treatment for cancer, their side effects on testosterone levels have not been as extensively explored.

Immune checkpoint inhibitors, which are intravenously administered therapies used to treat melanoma, include anti-programmed death-1 (anti-PD-1) antibodies (Pembrolizumab, Nivolumab) and anti-cytotoxic T-lymphocyte antigen-4 inhibition (anti-CTLA4) antibodies (Ipilimumab). There have also been recent developments of investigational therapies, including Indoximod and intratumoral SD-101. Intratumoral SD-101, a synthetic CpG oligonucleotide that stimulates Toll-like receptor 9 (TLR9) and is directly injected into the tumor site, has been shown to increase effectiveness of immune activation at the tumor site when used in combination with PD-1 inhibitors [6]. Indoximod, an inhibitor of the indoleamine 2,3-dioxygenase (IDO) pathway and is administered orally, has many effects on immune regulation allowing for the restoration of immune reactivity against cancer after there has been primary treatment with an active immunization processes (i.e., checkpoint inhibitor therapy) [7, 8]. The duration of treatment for unresectable and metastatic melanoma is currently recommended to continue until there is either disease progression or unacceptable toxicity, however, it is recommended to continue for approximately one year if used as an adjuvant therapy.

While side effects of immunotherapy have not been as comprehensively studied as older therapies, there are known adverse effects related to the attempted modulation of the immune system, including nonspecific inflammation and autoimmunity [9]. Studies over the last decade have begun documenting increasing reports of endocrine dysfunction following the initiation of immunotherapy. Specifically, hypophysitis, or inflammation of the pituitary gland, has been reported in 9–13% of patients treated with immunotherapy [3, 10–15]. Most of the patients who developed hypophysitis received Ipilimumab and there were higher rates observed in males (16.1%) than females (8.7%) [15–17]. Hypophysitis results in dysfunction of the pituitary gland and can cause a deficiency of one or more pituitary hormones, including luteinizing hormone (LH), which may then lead to testosterone deficiency. Thus far, no studies have specifically focused on androgen levels associated with immunotherapy or the clinical significance of androgen hormone deficiency. In our literature review, there was only one study which investigated testosterone levels following immunotherapy. The study reported incidences of Ipilimumab-associated low testosterone, in the absence of hypophysitis, in nine out of 256 patients assessed [15]. However, this study did not routinely assess testosterone levels and few measurements were actually obtained. Additionally, reassessment of testosterone levels was not always performed, so it is difficult to ascertain whether the low testosterone levels were transient or permanent.

Symptoms of low testosterone can be nonspecific and include fatigue, weight fluctuations, muscle loss, depression, insomnia, and sexual dysfunction [4, 18]. Sexual symptoms of androgen deficiency include decreased libido and erectile dysfunction [18, 19]. The implications of sexual dysfunction are far reaching, and may include reduction in self-esteem, impact on relationships, anxiety, and depression [4, 18]. It has been documented that male cancer survivors are likely to experience fatigue, impaired quality of life, and sexual dysfunction, regardless of type of cancer treatment received, and these effects are exacerbated by those with concurrent androgen deficiency [4, 5].

While the impact from chemotherapy agents on patients’ reproductive potential is well known, the impact from immunotherapy still needs to be established, as melanoma is becoming more prevalent in the AYA (adolescent and young adult) population [20–22]. Preclinical studies of Ipilimumab in monkeys demonstrated a clinically significant decrease in testicular weight, with no evidence of sperm histopathology changes [20]. A small study assessing testicular histopathology after immunotherapy for metastatic melanoma found evidence that spermatogenesis may be impacted by immunotherapies, however, the mechanism remains unclear [23]. While it is difficult to calculate fertility risk based on these small studies, there is concern for some level of gonadal dysfunction that needs to be further explored [24].

While immunotherapy has proven to be a successful treatment option for melanoma, there is concern for treatment-related dysfunction of the hypothalamic-pituitary-gonadal axis, which may impair quality of life [4, 5, 18, 20]. As immunotherapy is now widely used for a variety of cancers specifically in early stages, including non-small cell lung cancer, bladder cancer, renal cell carcinoma, and Hodgkin’s lymphoma [13], understanding the side effects associated with immunotherapy, while keeping in mind the direct impact of the treated malignancy, will allow for better recognition and earlier treatment for adverse effects in many future patients.

The aims of our study include: (1) detect the proportion of men who have low testosterone levels before, during, and or/after receiving immunotherapy for malignant melanoma; (2) identify risk factors for development of low testosterone levels during treatment with immunotherapy; (3) and determine the proportion of men who receive testosterone replacement therapy after detection of low testosterone levels.

## RESULTS

**Figure 1:**
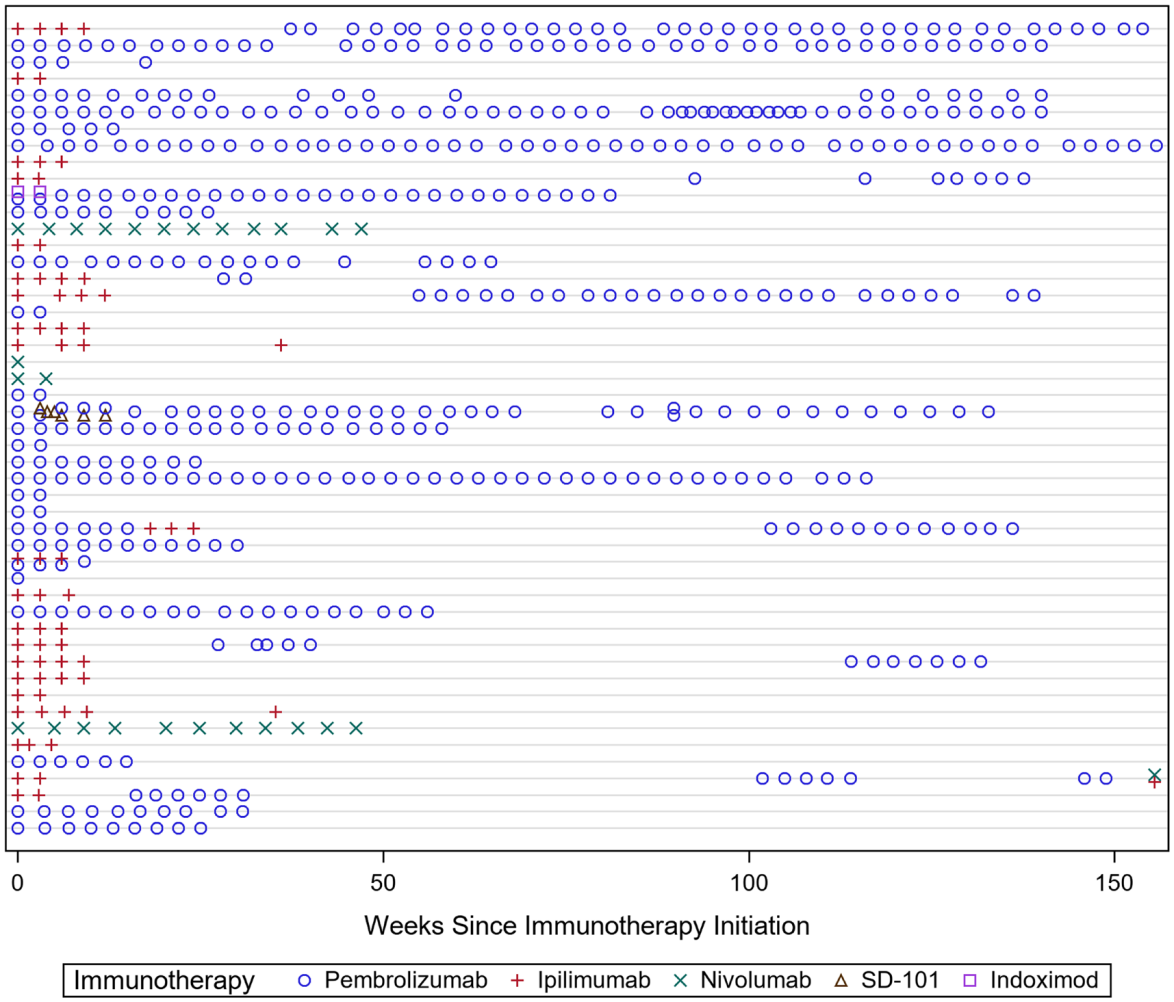
The different rows demonstrate each patient’s treatment paradigm across time. The heterogeneity in treatment regimens made it difficult to distinguish how each individual immunotherapy agent affected testosterone levels. On covariate analysis, there was no significant association between testosterone levels and treatment modality.

Forty-nine patients met inclusion criteria. Patient demographics are reported in Table 1. Median age at diagnosis was 64 (33–92) years. Fifteen patients had stage 3, 33 patients had stage 4, and one patient had a primary pineal gland melanoma that was not staged. At the time of analysis, 16 patients were deceased and 31 patients were alive, 13 of whom were tumor free. Two patients transferred care to a local cancer center and their status was unknown. Patients were treated with Pembrolizumab, Ipilimumab, Nivolumab, or investigational immunotherapy agents Indoximod and intratumoral SD-101 (Figure 1). Of the 49 patients who received immunotherapy, 45 of these patients had metastatic disease. The other four patients received immunotherapy as adjuvant therapy for melanoma.

**Table 1:** Patient demographics at time of diagnosis

Patient Demographics	*n*, %
Number of patients, male	49 (100%)
*Staging*:	
Stage 3	15 (30.6%)
Stage 4	33 (67%)
Primary pineal gland melanoma	1 (0.2%)
Previous history of melanoma	3 (6.1%)
Obesity	26 (53.1%)
Diabetes	10 (20.4%)
Heart disease (CAD, HTN)	35 (71.4%)
Hypothyroidism	8 (16.3%)
Obstructive sleep apnea	5 (10.2%)
Chronic kidney disease	3 (6.1%)
Corticosteroid use	1 (2.0%)
Opioid use	4 (8.2%)
Pre-existing autoimmune disease	2 (4.1%)
Previous cancer diagnosis	10 (20.4%)
*Type of cancer*:	
Basal cell carcinoma	2 (20.0%)
Bladder cancer	1 (10.0%)
Colon cancer	1 (10.0%)
Diffuse large B cell lymphoma	1 (10.0%)
Hodgkin’s lymphoma	1 (10.0%)
Neuroendocrine tumor	1 (10.0%)
Prostate cancer	3 (30.0%)
Received radiation therapy	26 (53.1%)
*Type of radiation*:	
Local	12 (46.2%)
Brain	14 (53.8%)
Enrolled in clinical trial	14 (28.6%)

**Figure 2:**
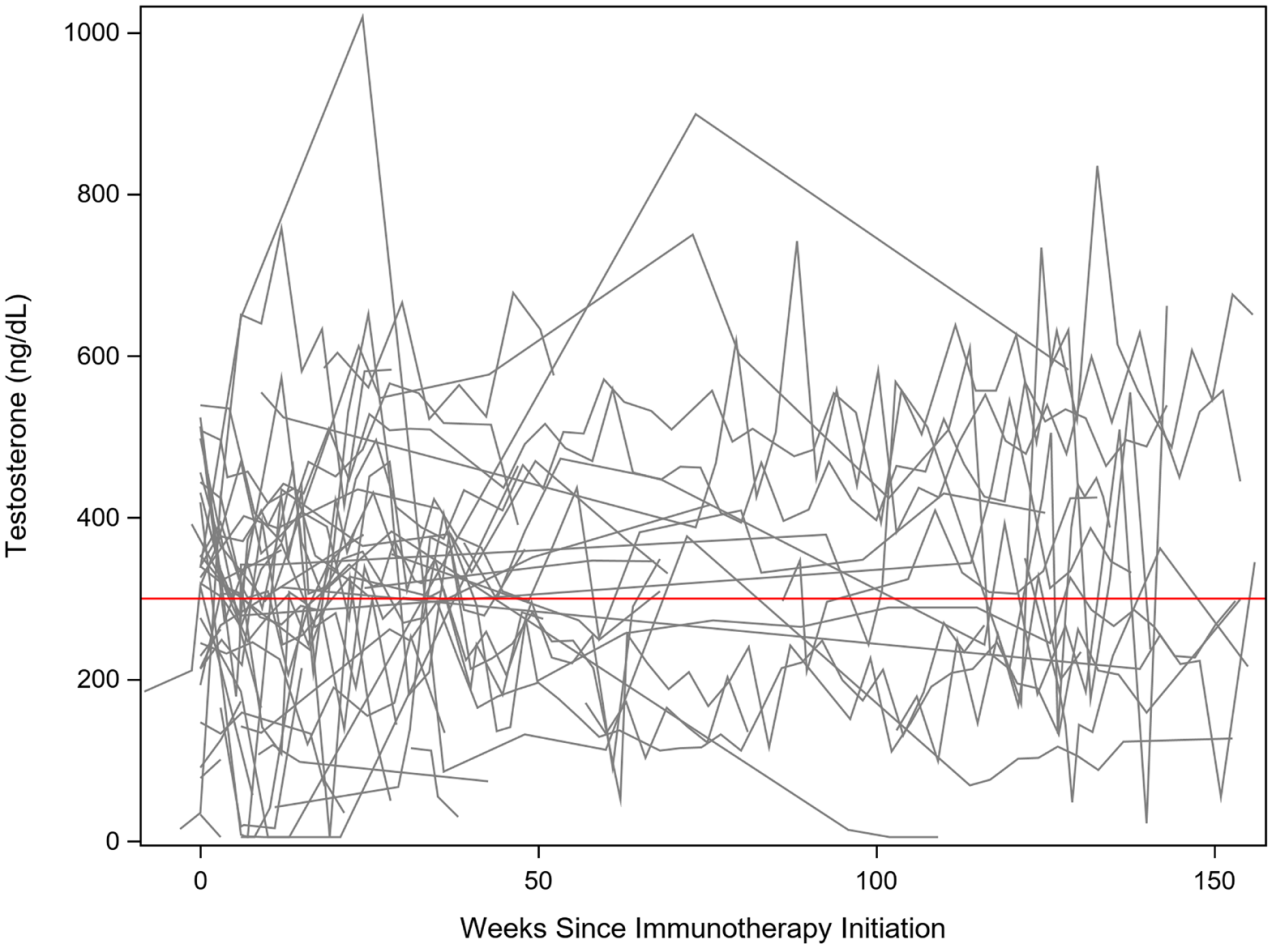
Testosterone levels assessed after initiation of immunotherapy. The red line is a reference for 300 ng/dL. There was large intrapersonal variability in testosterone levels, as demonstrated by the oscillating values within each patient. However, it is evident many patients had testosterone levels drop below 300 ng/dL, with many of those patients dipping well below 200 ng/dL.

Low testosterone was identified in 34 out of 49 patients at some point during treatment with immunotherapy (Figure 2). Of these 34 patients with low testosterone, eight had their testosterone levels return to > 300 ng/dL within three months after detection. One patient’s testosterone level returned to normal after one year. Twenty-five patients with low testosterone did not see a complete recovery in their testosterone levels throughout the remainder of their follow-up. Four patients developed hypophysitis and subsequent hypopituitarism, all of whom received Ipilimumab. All patients who developed hypophysitis began showing evidence of hypopituitarism after 2–3 doses of Ipilimumab. Forty-three patients reported fatigue to their provider during their treatment with immunotherapy. Only three patients, all diagnosed with hypopituitarism, were treated with testosterone replacement therapy. The fourth patient diagnosed with hypopituitarism was treated with corticosteroids. Ten (29%) of the patients who developed low testosterone had normal testosterone levels prior to initiation of immunotherapy (Table 2). Eight (24%) of the patients had a low baseline testosterone prior to immunotherapy initiation, and their levels remained low throughout their treatment. Thirteen (38%) of the patients identified with low testosterone did not have a baseline testosterone level checked prior to starting immunotherapy. Three (9%) of the patients had low baseline testosterone levels, however, they also had brain radiation within two months prior to checking their baseline level.

**Table 2:** Additional clinical information of the patients with previously normal testosterone who subsequently developed low testosterone after treatment with immunotherapy

Clinical information of patients with normal T who developed low T after immunotherapy									
Pt	Age at diagnosis	Immunotherapy Agent(s)	Baseline T	Lowest T value	Resolution of low T	Comorbidities	Radiation	Clinical trial	Endocrine dysfunction during tx
1	50	Ipilimumab, Pembrolizumab	Normal (314)	55	No	Heart disease	Local	-	-
2	72	Ipilimumab	Normal (498)	78	No	-	-	-	-
3	66	Pembrolizumab	Normal (319)	144	No	Heart disease	Brain	-	Pituitary, adrenal
4	61	Ipilimumab, Pembrolizumab	Normal (338)	69	No	Obese, heart disease, diabetes, hypothyroid	Brain	Yes	-
5	82	Pembrolizumab	Normal (399)	181	No	Heart disease, diabetes	-	-	-
6	59	Ipilimumab, Pembrolizumab	Normal (419)	< 5	No	Obesity, heart disease	Local	-	Hypophysitis
7	35	Ipilimumab, Pembrolizumab	Normal (524)	170	Yes	Obesity	Brain	Yes	Pituitary, thyroid
8	85	Pembrolizumab	Normal (351)	206	Yes	Obesity, heart disease, previous prostate cancer	-	-	Pituitary, adrenal
9	46	Pembrolizumab	Normal (369)	22	Yes	-	-	-	Adrenal
10	52	Pembrolizumab	Normal (456)	133	Yes	Obesity, heart disease, OSA, previous melanoma	Local	-	Pituitary, adrenal, and thyroid
**Avg:**	**60.8**		**398.7**	**105.8**					

While these patients had diverse treatment courses, most of these patients had multiple comorbidities prior to their development of low testosterone.

Covariate analysis demonstrated no significant association between low testosterone and treatment with either Ipilimumab, Pembrolizumab, or Nivolumab. On univariate analysis, obesity and enrollment in a clinical trial were associated with lower testosterone levels (Table 3). During the treatment course with immunotherapy, 19 patients had evidence of pituitary dysfunction, 16 patients had evidence of adrenal dysfunction, and 18 patients had evidence of thyroid dysfunction. On univariate analysis, a trend was noted between testosterone and adrenal dysfunction and thyroid dysfunction. On multivariable analysis (Table 4), participation in a clinical trial and weeks since immunotherapy initiation were significant (*p* < 0.01), and a trend was noted for thyroid dysfunction (*p* = 0.08) and adrenal dysfunction (*p* = 0.08). More specifically, clinical trial patients had testosterone levels that were 80.38 ng/dL lower on average. There was not a clinically significant difference between clinical trial enrollment and cancer stage. Of the 15 patients with stage 3, five were enrolled in a clinical trial; while there were nine out of 33 patients with stage 4 enrolled in a clinical trial. Testosterone levels declined at a rate of 0.71 ng/dL per week, on average. Obesity, adrenal dysfunction, and thyroid dysfunction tended to be associated with lower testosterone levels.

**Table 3:** Univariate analysis assessing for associations with low testosterone and patient demographics, co-morbidities, treatment modalities, and treatment complications

Covariate	Univariate Analysis				
	Testosterone (ng/dL)				
	Level	Beta	95% CI		*P*-value
Obesity	Yes	–56.71	–110.41	–3.01	0.04
	No	Ref	-	-	
Diabetes	Yes	–18.77	–89.16	51.63	0.60
	No	Ref	-	-	
Heart Disease (CAD, HTN)	Yes	–16.97	–78.93	44.99	0.59
	No	Ref	-	-	
Cancer Stage	4	–18.05	–81.07	44.97	0.57
	3	Ref	-	-	
Other Cancer Diagnoses	Yes	40.46	–27.45	108.37	0.24
	No	Ref	-	-	
Clinical trial	Yes	–87.04	–143.19	–30.88	< 0.01
	No	Ref	-	-	
Radiation	Yes	–17.80	–65.16	29.56	0.46
	No	Ref	-	-	
Radiation	Local	–2.81	–63.67	58.04	0.56
	Brain	–34.24	–97.67	29.20	
Pituitary	Yes	–22.01	–64.11	20.08	0.30
	No	Ref	-	-	
Adrenal	Yes	–45.52	–96.51	5.47	0.08
	No	Ref	-	-	
Thyroid	Yes	–40.99	–86.22	4.23	0.08
	No	Ref	-	-	
Testosterone Replacement	Yes	98.91	–11.35	209.17	0.08
	No	Ref	-	-	
Age	Units = 1	0.64	–1.35	2.63	0.52
BMI at diagnosis	Units = 1	–4.22	–9.80	1.36	0.14
Months Since Diagnosis	Units = 1	–2.13	–5.02	0.75	0.14
Time obtained (minutes)	Units = 60	0.06	–2.81	2.93	0.97
Weeks Since Immunotherapy Initiation	Units = 1	0.44	–0.11	0.99	0.12

Obesity and enrollment in a clinical trial were associated with low testosterone. A trend is observed between low testosterone and adrenal and thyroid dysfunction.

**Table 4:** Multivariable model was utilized to include variables that were significant or nearly significant on univariate analysis

Multivariable analysis					
Covariate	Testosterone (ng/dL)				
	Level	Beta	95% CI		*P*-value
Intercept		348.53	300.37	396.69	**< 0.01**
Obesity	Yes	–36.72	–90.57	17.13	0.18
	No	Ref	-	-	
Clinical trial	Yes	–80.35	–139.85	–20.86	**< 0.01**
	No	Ref	-	-	
Adrenal	Yes	–50.54	–108.60	7.53	0.08
	No	Ref	-	-	
Thyroid	Yes	–43.84	–94.37	6.69	0.08
	No	Ref	-	-	
Testosterone Replacement	Yes	135.84	–109.79	381.48	0.14
	No	Ref	-	-	
Weeks Since Immunotherapy Initiation	Units = 1	0.71	0.18	1.25	**< 0.01**

Enrollment in a clinical trial and weeks since immunotherapy initiation were significant.

## DISCUSSION

In our cohort, roughly two-thirds of patients developed low testosterone at some point during their treatment with immunotherapy. While some patients did have a recovery in their testosterone levels after a few months, over half of the patients continued to have low testosterone levels throughout the remainder of their follow-up. Despite persistently low testosterone levels in 25 patients, only three patients were treated with testosterone replacement therapy, all of whom had hypophysitis and testosterone levels < 5 ng/dL. In addition to laboratory evidence of low testosterone, patients were also symptomatic as the vast majority of patients reported fatigue to their providers. The three patients who received testosterone replacement therapy reported improved energy levels after treatment, however, they also received steroids and thyroid replacement therapy so it cannot be determined from our study whether testosterone replacement directly improves energy in these patients. While many factors contribute to fatigue, low testosterone levels may be playing an important role in the development of fatigue in these cancer patients. There was no chart documentation of whether providers assessed for other symptoms of low testosterone, including low libido or sexual dysfunction. These findings suggest that providers may be under-diagnosing and under-treating low testosterone in melanoma patients receiving immunotherapy.

Out of our patient population, 8% developed hypophysitis. All four patients showed evidence of hypopituitarism within three months of initiation of Ipilimumab, which has been documented in previous studies [15–17]. These data suggest that patients being treated with Ipilimumab need diligent monitoring of their endocrine function during the first three months of their treatment and up to 12 months after completion of their immunotherapy treatment [25].

In our study, there were clinically significant associations of low testosterone with obesity and patients that were enrolled in a clinical trial. It has been well documented that obesity is associated with low testosterone due to the high expression of aromatase in adipocytes, which converts testosterone to estradiol and lowers circulating androgens [26]. Of the 14 patients enrolled in a clinical trial, 10 of these patients developed low testosterone. The type of clinical trial each patient participated in was extremely variable, however, Indoximod and high dose (10 mg/kg) Ipilimumab were utilized most frequently. These patients may have been more likely to develop lower testosterone levels due to the higher dose of immunotherapy and the use of investigational immunotherapy agents, the side effects of which have not been completely studied. Additionally, Indoximod was used in conjunction with an anti-PD1 immunotherapy agent. The use of two immunotherapy agents has typically shown to have a synergistic effect on cancer control. This synergy may also reflect how immunotherapy affects testosterone levels, and therefore, those patients who are receiving two types of immunotherapy could be more susceptible to developing low testosterone. There may have also been a delayed effect of prior therapeutic agents used to treat melanoma on testosterone levels. On multivariable analysis, there was also an association with decreasing testosterone levels and weeks since initiation of immunotherapy. On average, the testosterone levels declined at a rate of 0.71 ng/dL per week. This change is minimal and most likely not clinically significant.

To our knowledge, this is the first study directly analyzing the effect of immunotherapy on testosterone levels in patients with melanoma. One limitation of this study is the low number of analyzed patients. While there were 143 male patients with malignant melanoma that received immunotherapy from 2009–2019, only 49 patients had at least two testosterone levels drawn. There were also inconsistencies as to when these patients had their testosterone levels checked. Only 30 of the 49 patients had a baseline testosterone level checked prior to initiation of immunotherapy. The other 19 patients had their level checked some variable amount of time after receiving immunotherapy. Additionally, further assessments of testosterone levels were not at regular intervals. Because roughly half of the patients had irregular assessment of testosterone levels, we are unable to report the causality of low testosterone.

Another limitation of this study is the heterogeneity within each patient’s treatment course, which made it difficult to analyze patients based on specific treatment received. Specifically, of the 34 patients identified with low testosterone during immunotherapy treatment, two patients were also being treated with steroids and five patients received concurrent radiation therapy (one received retroperitoneal radiation while the other four received brain radiation). It cannot be determined whether the low testosterone was due to immunotherapy and/or the concurrent treatment with steroids or radiation therapy.

As this was a retrospective study, fertility was not assessed in these patients. However, as melanoma is becoming more prevalent in the AYA population, future work needs to be completed to addresses any potential fertility risk in melanoma patients receiving immunotherapy, with particular attention to sperm count and function.

In conclusion, patients with stage 3 or 4 melanoma treated with immunotherapy appear to be at an increased risk of developing testosterone deficiency during their treatment. The majority of patients also report fatigue, which is commonly associated with low testosterone levels, yet only a minority of those with low levels received testosterone supplementation. Patients who are obese and have been enrolled in a clinical trial appear to be at increased risk of developing low testosterone during treatment with immunotherapy, and these patients would benefit from close observation and potentially testosterone replacement therapy. Encouraging weight loss in obese patients diagnosed with low testosterone should also be considered in conjunction with testosterone replacement therapy. Regardless of the reason for the development of low testosterone, our study has identified that this patient population is at risk for under-diagnosis and under-treatment of low testosterone. Further research needs to be performed to assess whether or not testosterone supplementation improves patient-reported outcomes in this patient population.

## MATERIALS AND METHODS

**Figure 3:**
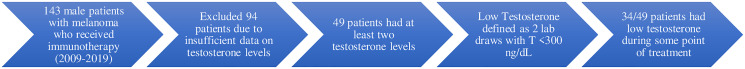
Systematic review of 149 patients. Patients were excluded in the study if they had fewer than two assessments of testosterone levels.

After seeking Institutional Review Board and Ethics approval, we performed a retrospective chart review of patients with malignant melanoma treated with immunotherapy at Holden Comprehensive Cancer Center from 2009–2019 using the Melanoma Molecular Epidemiology Resource database. Patients were included in the analysis if they had at least two testosterone levels checked while on immunotherapy (Figure 3). Low testosterone was defined in this study as total testosterone < 300 ng/dL upon two consecutive lab draws, according to American Urological Association (AUA) guidelines on testosterone deficiency [27]. Blood was collected and total testosterone was assessed via electrochemiluminscence immunoassay.

Patients were also evaluated for pituitary, adrenal, and thyroid dysfunction, based on the reference ranges from Clinical Laboratory Improvement Amendments certified laboratory at the University of Iowa Hospitals and Clinics. Pituitary dysfunction was defined as adrenocorticotropic hormone (ACTH) < 7 pg/mL. Adrenal dysfunction was defined as cortisol < 3 μg/dL. Thyroid dysfunction was defined as TSH < 0.27 or > 4.20 μIU/mL. Patient demographics and co-morbidities were gathered from chart review. Obesity was classified as BMI > 30 at the time of initial office visit.

Mixed effects regression models were applied to assess for changes in serum testosterone levels over time while adjusting for potential confounders. Time-dependent covariates were incorporated to capture changes in treatment and onset of other medical conditions. Random effects were included to account for the longitudinally correlated nature of repeat assessment and unequal time spacing between visits. Beta coefficients and 95% confidence intervals are reported. All statistical testing was two-sided and assessed for significance at the 5% level using SAS v9.4 (SAS Institute, Cary, NC). Power calculations were not performed, as this was a retrospective study meant to gain preliminary estimates of the true underlying population values.
